# Asthma and the microbiome: *defining the critical window in early life*

**DOI:** 10.1186/s13223-016-0173-6

**Published:** 2017-01-06

**Authors:** Leah T. Stiemsma, Stuart E. Turvey

**Affiliations:** 1 Department of Microbiology & Immunology, University of British Columbia, Vancouver, BC Canada; 2 BC Children’s Hospital, Vancouver, BC Canada; 3 Department of Pediatrics, University of British Columbia, Vancouver, BC Canada; 4 Department of Pediatrics, BC Children’s Hospital, 950 West 28th Avenue, Vancouver, BC V5Z 4H4 Canada

**Keywords:** Microbiota, Asthma, Early life, Critical window, Hygiene hypothesis, Microflora hypothesis

## Abstract

Asthma is a chronic inflammatory immune disorder of the airways affecting one in ten children in westernized countries. The geographical disparity combined with a generational rise in prevalence, emphasizes that changing environmental exposures play a significant role in the etiology of this disease. The microflora hypothesis suggests that early life exposures are disrupting the composition of the microbiota and consequently, promoting immune dysregulation in the form of hypersensitivity disorders. Animal model research supports a role of the microbiota in asthma and atopic disease development. Further, these model systems have identified an early life critical window, during which gut microbial dysbiosis is most influential in promoting hypersensitivity disorders. Until recently this critical window had not been characterized in humans, but now studies suggest that the ideal time to use microbes as preventative treatments or diagnostics for asthma in humans is within the first 100 days of life. This review outlines the major mouse-model and human studies leading to characterization of the early life critical window, emphasizing studies analyzing the intestinal and airway microbiotas in asthma and atopic disease. This research has promising future implications regarding childhood immune health, as ultimately it may be possible to therapeutically administer specific microbes in early life to prevent the development of asthma in children.

## Background

Recent evidence supports a role of the intestinal microbiome in the development of childhood asthma and atopic disease. Animal model studies have made significant advancements in the quest to understand the gut-lung axis; identifying large-scale shifts in gut microbial compositions in asthma and allergy-induced mice and manipulating the intestinal microbiome with antibiotics, which enhanced the severity of these diseases [1–3]. However, with the advancement of DNA and RNA sequencing technologies and the establishment of large longitudinal human birth cohorts, it is becoming clearer that gut microbial dysbiosis in human atopic diseases is characterized not by global changes to the composition of the intestinal microbiota, but by taxa-specific shifts in abundance at the family, genus, and even species’ levels [4–6]. Perhaps unsurprisingly, these taxa-specific changes are most prominent within the first 100 days of life, during which the human immune system is most plastic in its development [4–6]. Given these scientific developments, this review aims to provide an overview of the recent advances in human microbiome research in asthma and atopic disease.

## The global burden of asthma and atopic disease

Allergic asthma is an immunoglobulin E (IgE)-mediated chronic inflammatory disease of the airways [7]. Other manifestations of IgE-mediated or “atopic” diseases include: atopic dermatitis (also referred to as eczema), allergic rhinitis, and food allergy [8]. These diseases typically manifest in early childhood and can be chronic lifelong burdens for many people. However asthma is often viewed as the most burdensome atopic disorder, due to the prevalence (235 million people worldwide) and associated mortality (an estimated nine asthma-related deaths per day in the United States) [9, 10]. Asthma has become the most prevalent childhood disease in recent decades, affecting approximately one in ten children worldwide [8]. Aside from the obvious danger associated with asthma, this disease is very disruptive to a normal daily lifestyle for children, and is the leading cause of emergency room visits and absenteeism from school [11].

Some of the most striking data related to asthma prevalence comes from the United States, where it was reported from 1999 to 2009, that the proportion of people diagnosed with asthma increased by 15% [10]. In other Westernized countries (e.g. Canada, Australia, and the UK) the prevalence of this disease was reported to be even higher (up to 30% in some countries), while many countries in Eastern Europe and Asia report a much lower prevalence of this disease (~5%) [8, 12, 13]. This rapid increase in prevalence of asthma (and other atopic diseases) as well as the apparent geographical disparity suggests an etiology that is more complex than population genetic variation.

## ‘The post-industrial epidemic’

The underlying cause of asthma is a complex product of genetic and environmental factors resulting in significant heterogeneity of the disease. Parental history of asthma increases the likelihood of developing this disease, however assessment of this factor alone is not enough to confirm a person’s risk of asthma development [14–16]. There is also evidence of a strong link between sex and increased risk of asthma development in children, as boys are more likely to develop childhood asthma than girls [17, 18]. Further, genome-wide-association-studies have identified candidate genes that play a role in asthma susceptibility (*ORMDL3* and *SMAD3*) [19]. Thus it is clear that human genetics contribute to asthma pathogenesis, however the rapid rise in asthma prevalence suggests changing environmental factors are biasing the developing human immune system toward these hypersensitivity diseases [20].

In addition to the within-generation rise in the prevalence of asthma, there is also an inverse relationship between the incidence of infectious diseases and hypersensitivity diseases, where a high incidence of infectious diseases appears to protect against allergic and autoimmune diseases [21]. Further, the geographical disparity of asthma and atopic diseases is shifting, as developing countries become industrialized and their living conditions become more like the Western world [22]. Thus it appears that there may be a link between the development of hypersensitivity diseases and the urbanization or modernization of society [23]. Many urban environments have similar characteristics (lower air quality, higher population density, lower economic status) that predispose populations to asthma; and similar to the geographical disparity of this disease, rural areas with comparable environments do report greater incidences of hypersensitivity diseases [24, 25]. There is also the possibility that urbanization does not support optimal immune development due to a decrease in exposure to environmental microbes as humans shift from an outdoor lifestyle to a more indoor lifestyle that is characteristic of urban societies [26].

This concept of decreased microbial exposure in modern or more urban societies has become a booming research area in the etiology of immune dysregulation. One particular arm of asthma etiology in particular, focuses on factors associated with improved health and hygiene; for example, increased antibiotic exposure, and household size [23, 27–35]. In particular, David Strachan extensively studied the relationship between household size and atopic disease in the late 1980s, and his initial findings led him to propose the hygiene hypothesis of allergic disease in 1989 [36]. This hypothesis sets the stage for the current analyses assessing the role of microbial exposure in the development of asthma and other hypersensitivity disorders.

## External and internal microbes as protectors against asthma

### The hygiene hypothesis

The hygiene hypothesis proposes that a lack of early life exposure to microbes alters early life immune system priming and, consequently, increases susceptibility to atopic diseases [20]. Strachan theorized that older siblings promote increased exposure to environmental microbes through inevitable unhygienic contact, which results in decreased likelihood of atopic disease development in younger siblings [36]. He supported his hypothesis by showing that household size was inversely correlated with the development of hay fever (i.e. allergic rhinitis) in a cohort of 11,765 children [37]. Since Strachan’s original proposal, the hygiene hypothesis has expanded to include additional environmental factors (such as mode of birth, antibiotic exposure, household pets, etc.), which also alter the microbial exposure of infants [33–35]. Further, with substantial improvements in genetic sequencing technology, the role of indigenous microbes has also been added to the mix.

### The microflora hypothesis

The microflora hypothesis extends Strachan’s hygiene hypothesis by emphasizing the role of microbes residing in and on the human body (collectively known as the human microbiota) [38]. Originally, these microbes were considered to be commensal, having little effect on human physiology [38]. However they have since been implicated extensively in human health and development, and it is clear that there is a microbial-immune cell interface, in which cross-talk between microbes and immune cells aids in the development of immune tolerance [39–42]. Notably, the focus of this hypothesis is the gastrointestinal tract, one of the most populated zones of the human body [38]. It proposes that perturbations to the colonization and composition of the intestinal microbiota (dysbiosis), disrupts this natural microbe-immune cell interface, biasing the developing infant immune system toward a hyper-sensitive (allergic) state [38, 43]. In support of the microflora hypothesis, a recent study found that uncontacted Amerindians (indigenous peoples of the Americas) exhibited higher levels of bacterial and functional diversity in their skin and fecal microbiota than any other human population previously reported, suggesting that modern societal practices (perturbations) have strong implications in the development of the microbiota [44]. Regarding the role of the intestinal microbiome in asthma, the gut-lung axis attempts to explain the mechanisms guiding gut microbe-lung immune cell cross talk.

## The gut-lung axis

### Innate immunity microbial crosstalk

The gut-lung axis attempts to mechanistically define how microbes in the gut might influence immune function in the lung [45]. One potential connection is through interactions of the gut microbiota with pattern recognition receptors of the innate immune system [46]. It is well established that pathogen-associated molecular patterns (PAMPs) such as lipopolysaccharide (LPS), CpG, and peptidoglycan can stimulate Toll-like receptor (TLR) signaling, which confers downstream activation of many genes that regulate inflammation and innate immune responses [47]. Similar to the antigen-recognition and IgE-mediated hypersensitivity pathways, dendritic cells (DCs) are also the intermediaries of gut microbiota-immune cell cross talk, as they regularly sample gut microbes in the intestinal lumen or lymphoid tissues [46]. DC sensing of gut microbiota PAMPs promotes immune tolerance in the intestine, but also results in phenotypic changes to DCs and migration to the mesenteric lymph node (MLN) to promote T cell priming [48]. In the MLN, T cells also acquire homing molecules (e.g. CCR4, CCR6), which initiate migration to other parts of the body, including the respiratory mucosa [49].

Thus it is possible that interactions with specific gut microbes, via their corresponding PAMPs, could result in varying phenotypic changes in DCs, with downstream effects on lymphocyte priming/homing and ultimately, shifts in anti-inflammatory responses in the airways [49]. In a house dust mite (HDM) model of allergic inflammation, chronic intranasal exposure to endotoxin (bacterial LPS) has been shown to protect mice from HDM-induced asthma [50]. The proposed mechanism of this protection is through A20 (ubiquitin modifying enzyme)-mediated inhibition of HDM-induced recruitment of conventional DCs to the lungs and mediastinal lymph nodes [50]. Further, prior 2-week treatment of mice with LPS suppressed proliferation and differentiation of adoptively transferred CD4+ HDM-specific 1-DER T cells in the mediastinal lymph nodes into IL-5 and IL-13—secreting T-helper (Th)-2 cells, highlighting the T cell priming effects of these DCs [50]. Though this is not a gut microbiota mediated pathway, it does highlight the ability of bacterial PAMPs (specifically LPS) to alter DC recruitment to the lungs and protect mice against asthma symptoms.

### Role of microbial-derived metabolites—short chain fatty acids

Another area of gut-lung axis research involves microbial-derived metabolites, such as short chain fatty acids (SCFAs). SCFAs are direct by-products of bacterial fermentation of carbohydrates and are key energy sources for many host tissues and gut bacterial species [51]. There are three major SCFAs, acetate, propionate, and butyrate, which are present in a molar ratio of 60:20:20, respectively [51]. These metabolites are known to modify gene expression through inhibition of histone deacetylases (HDACs), cytokine and chemokine production, and cell differentiation, proliferation, and apoptosis [52]. With regard to immune tolerance and inflammatory mechanisms, butyrate and propionate induce extrathymic T-regulatory (T-reg) generation through direct interactions with T cells and indirect interactions through DCs, potentially through the inhibition of HDACs [53]. Clostridial species are prominent SCFA producers, and butyrate production by these particular bacteria was associated with the generation of peripheral T-reg cells in the colon [54]. In a HDM-mouse model of experimental asthma, both acetate and propionate were capable of reducing cellular infiltration into the airways after HDM exposure [55]. Systemic propionate treatment modified bone marrow hematopoiesis and enhanced the generation of DC and macrophage precursors and subsequent recruitment of DCs less effective in promoting Th-2 cell polarization in the lungs [55]. In a later study using the same asthma mouse model, maternal intake of acetate was shown to reduce allergic airways disease in the adult offspring of mice [56]. Notably, both these studies initially assessed the role of a high fiber diet on the production of SCFAs and colonization of intestinal bacteria—highlighting the influence of diet, mediated by gut microbial changes, on the development of the immune system [55, 56]. The latter study, however, emphasizes intrauterine effects on the infant immune system, mediated by maternal diet, suggesting the need to consider prenatal prevention strategies using these gut microbial metabolites [56].

### Microbial influences on epigenetics

It is also possible that the intestinal microbiota is linked to lung immunity through microbe-mediated epigenetic modification. Distinct whole blood DNA methylation patterns were associated with two major bacterial phyla, either Firmicutes or Bacteroidetes and pathway analysis revealed differential methylation (associated with a high or low Firmicute/Bacteroidetes ratio) among genes enriched in functional networks such as cardiovascular disease, inflammatory responses, obesity, and lipid metabolism [57]. Further, production of bacterial methyl groups, cofactors (e.g. folate), and enzymes (e.g. methyltransferases) can both directly and indirectly affect host DNA methylation, and consequently may bias cell differentiation toward or against an immune profile that confers tolerance [58, 59]. There is also evidence that early life farm microbial exposures may influence the methylation of genes related to asthma and allergies [60, 61]. Lastly, reinforcing the age-sensitive role of the intestinal microbiota in hypersensitivity diseases, the presence of a conventional gut microbiota in previously germ-free (GF) neonatal (but not adult) mice decreased hypermethylation of *CXCL16*, which in turn decreased accumulation of invariant natural killer T (iNKT) cells (prominent in the pathogenesis of asthma) in the colon [41]. Thus ultimately, there is much more to learn regarding the mechanisms of the gut-lung axis in asthma and other lung disorders. Current research in asthma and atopic disease centers around how, mechanistically, the intestinal microbiota is linked to these disorders and whether early life changes to the intestinal microbiome can be therapeutically manipulated to promote immune tolerance.

## The intestinal microbiota in asthma and atopic disease

Mechanistic studies analyzing how the intestinal microbiome is involved in asthma and atopic disease are typically conducted in mouse models of allergic inflammation. GF mice lacking a microbiota show increased allergic responses, including increased lymphocyte and eosinophil inflammation in the airways, accompanied by increased Th-2 cytokines and elevated IgE production [62]. However many animal studies also focus on roles of distinct bacterial taxa in atopic disease development. In an ovalbumin (OVA)-model of asthma, oral supplementation of mice with two types of *Lactobacillus* showed that protection from allergic responses is mediated by specific bacterial species [63]. Supplementation with live *Lactobacillus reuteri* resulted in decreased airway hyper responsiveness, while treatment with *Lactobacillus salivarius* had no effect on the allergic symptoms of the mice [63]. This species-specific effect was also shown using three bacterial species, *Bifidobacterium longum*, *Bifidobacterium breve* and *L. salivarius* [1]. *L. salivarius* had no effect, and both *Bifidobacterium* species increased Peyer’s patch and splenic Foxp3+ T-reg cells in infant mice [1]. However, only *B. longum* introduced in the perinatal period resulted in T-reg cell induction in adult mice and protected against allergic airway inflammation in OVA-sensitized mice [1]. Notably, the age-sensitive induction of T-regs in adult mice by *B. longum* suggests the presence of an early life window in which microbial-driven immune changes are most effective.

Antibiotics (which disturb the intestinal microbiota composition) have also been shown to increase airway inflammation in mouse models of experimental asthma [2, 64, 65]. One study showed that combined oral antibiotic treatment of mice resulted in increased allergic inflammation, characterized by increases in serum IgE and circulating basophils [65]. Conventionally raised mice showed decreased proliferation of bone-marrow resident basophil precursors compared to the antibiotic treated mice, suggesting that these shifts in immune cells were mediated by alterations to the microbiota [65]. Thus collectively, these mouse model studies show that gut microbial alterations can result in changes in lung function, but it is becoming clearer through improved mouse-models and longitudinal human cohort research, that these microbe-mediated changes in immune development are most effective in early infancy.

## Mouse studies suggest an early life critical window

**Fig. 1:**
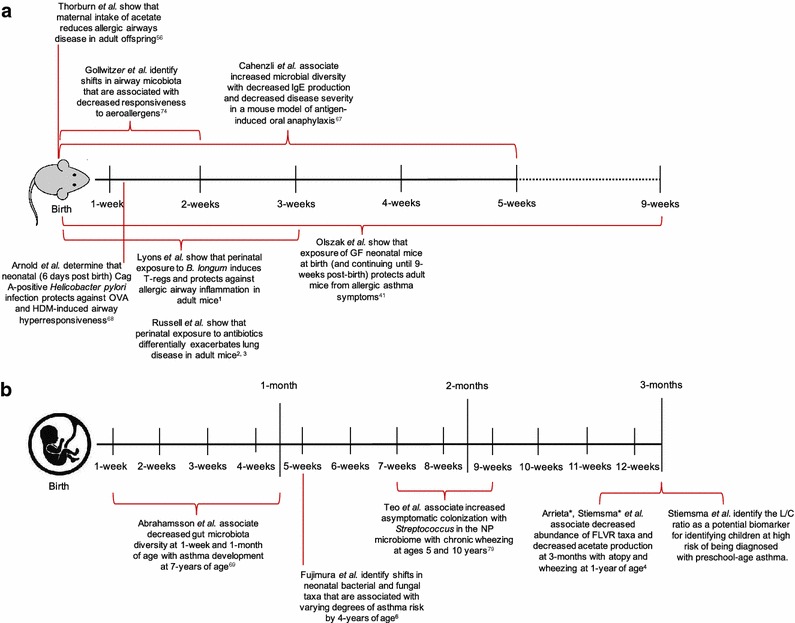
Defining the critical window of early life in **a** mice and **b** humans

Age is the main driver of compositional and functional differences in the intestinal microbiota [4, 66]. Thus it is perhaps unsurprising that many mouse studies assessing the role of the gut microbiota in atopic disease, find the results to be time sensitive (Fig. 1a). Cahenzli et al. demonstrate that global shifts in the composition of the intestinal microbiota (increased microbial diversity) in early life is required to regulate IgE production and decrease disease severity in a mouse-model of antigen-induced oral anaphylaxis [67]. In OVA- and HDM-models of allergic airway inflammation, oral infection with CagA-positive *Helicobacter pylori* resulted in protection against OVA and HDM-induced airway hyper responsiveness [68]. However, this bacterium-mediated protection against asthma was more apparent in mice infected neonatally compared to mice infected as adults [68]. As noted in the previous section (microbial influences on epigenetics), neonatal (but not adult-life) exposure to a conventional microbiota in GF OVA-challenged mice abrogated iNKT cells in the lungs and reduced serum IgE, proinflammatory cytokine levels, and eosinophilia in the bronchoalveolar lavage fluid, protecting mice from developing allergic asthma symptoms [41].

Russell et al. demonstrate the effects of early life antibiotic exposure in mice [2, 3]. This group showed that perinatal (in utero and up to 21 days after birth until weaning) versus strictly prenatal (in utero) vancomycin treatment of OVA-challenged mice exacerbates asthma-related immune responses [2]. Further, perinatal treatment of mice with another antibiotic, streptomycin, exaggerated lung inflammation in a Th-1/Th-17-driven model of hypersensitivity pneumonitis [3]. Notably, each antibiotic promoted expansion of specific bacterial phyla; streptomycin promoted expansion of Bacteroidetes, while vancomycin promoted expansion of Firmicutes [3]. This highlights both the selective effects of antibiotics on gut microbial taxa and the ability of an antibiotic-altered microbiota to differentially enhance disease susceptibility to specific lung diseases [3, 64]. Notably, all of these studies highlight a critical window (from birth to weaning in mice) in which microbial alterations can promote or protect against asthma and atopic diseases. However until recently, this critical window was not characterized in humans.

## Longitudinal human studies define the early life critical window

Mouse studies have made substantial mechanistic strides in microbiome-atopic disease research. In parallel, improvements in DNA sequencing technology over the past decade have made analyzing the microbiomes of humans much more feasible. With these sequencing improvements, a similar ‘critical window’ in which microbial alterations can be associated with the development of asthma and atopic disease is also becoming more apparent in humans (Fig. 1b). Using 454-pyrosequencing, one study associated changes in gut microbial diversity at 1-week and 1-month of age with asthma development at school age [69].

However more recent studies have identified shifts in specific bacterial taxa in early life, rather than global compositional changes, that are associated with increased risk of asthma later in life. In fact, our group identified decreases in the abundances of four bacterial genera, *Faecalibacterium, Lachnospira, Rothia, and Veillonella* (FLVR), in the 3-month fecal microbiota, which were associated with atopy and wheezing at 1-year of age among 319 infants enrolled in the Canadian Healthy Infant Longitudinal Development (CHILD) Study [4]. Atopy and wheezing are clinically used to predict asthma development in children, and subjects positive for both atopy and wheezing were most likely (compared to wheeze only, atopy only, and control subjects) to develop asthma by 3-years of age—suggesting that these early life genera shifts are associated with increased risk of asthma development [4]. Further, these four bacterial taxa ameliorated asthma in an OVA-challenged mouse model, supporting their immune-modulatory roles in protecting against asthma development [4].

Since the CHILD Study is a longitudinal cohort, our group was able to conduct a follow-up study on this same cohort when they reached 4-years of age and could be diagnosed with preschool-age asthma [5]. We found that *Lachnospira* remained decreased in the 3-month fecal microbiota while one particular bacterial species, *Clostridium neonatale*, was increased in asthmatics at this time-point [5]. Demonstrating the diagnostic potential of these particular microbes, we calculated a ratio of *Lachnospira* to *C. neonatale* (L/C) and using quartile analysis, showed that children with the lowest L/C ratio (quartile 1) were 15 times more likely to be diagnosed with preschool-age asthma than children in the other L/C quartiles [5]. Most interestingly, however, both of these studies identified these gut microbial changes in the first 3 months of life only, highlighting this time frame as the early life critical window during which gut microbial dysbiosis is most influential in promoting asthma and atopic disease in humans [4, 5].

Notably however, additional bacterial taxa as well as other microbes (e.g. fungi) have been associated with asthma and atopic disease development in children [6, 70]. In fact, a recent study published in *Nature Medicine* was able to distinguish asthmatic and atopic children by their neonatal (35 days post birth) intestinal microbiome compositions [6]. Children in the highest risk group showed shifts in specific bacterial and fungal taxa, highlighting roles of various gut microbes in human immune development, which are identifiable even earlier than 3-months of age [6]. Thus, even now it is becoming more evident that; (i) there are likely many other gut microbes associated with asthma and atopic disease development in humans; and (ii) that the ‘critical window’ for identifying these gut microbial shifts in humans could be even smaller than 100 days post birth. Further, in an effort not to overlook a potentially obvious link between the microbiome and airway inflammation, recent studies have identified associations and mechanistic links between airway microbes and asthma and atopic disease development.

## Role of the airway microbiota

Though the intestinal microbiome is one of the most populated regions of the human body, recent research supports a role of the airway microbiome in asthma and atopic disease pathogenesis. In an OVA-induced mouse model of asthma, administration of a common gut pathogen, *E. coli*, to the lung was shown in a TLR4-dependent manner to induce γδ-T cells, decrease activation of lung DCs, and abrogate Th-2 cytokine production to confer protection of mice from allergic airway inflammation [71].

In humans, airway microbial dysbiosis has been associated with increased risk of asthma [72, 73]. 16S rRNA analysis of sputum samples showed higher bacterial diversity and increased abundance of Proteobacteria in asthmatic adults compared to non-asthmatic adults [73]. Another adult study analyzing the bronchial microbiota was able to identify differences in microbial composition associated with asthma severity [72]. When compared to healthy controls, severe asthmatics were enriched in Actinobacteria and *Klebsiella* species [72]. However compared to patients with moderate asthma, patients with severe asthma were enriched in many Actinobacterial taxa and showed decreased abundances of Proteobacteria [72].

Continuing the early life theme, Gollwitzer et al. provide evidence of a 2-week window in which shifts in the airway microbiota are associated with decreased responsiveness to aeroallergens and the induction of Helios^−^ T-regs in a programmed death ligand 1 (PD-L1)-mediated manner [74]. If PD-L1 is blocked only in the first two weeks of life and allergic airway inflammation is induced after 4-weeks of age, the exaggerated allergic airway inflammation in neonatal mice is maintained to adulthood [74]. Further, in a study of 234 human children, researchers associated early (7–9 weeks post-birth) asymptomatic colonization with *Streptococcus* in the nasopharyngeal (NP) microbiome with chronic wheezing at ages 5 and 10 years [75]. Interestingly, they also suggest the NP microbiome as a determinate for the spread of respiratory infections to the lower airways, which are also significant risk factors for asthma development [75]. Thus there may be specific early life non-pathogenic airway microbes associated with asthma, but it is also possible that dysbiosis in the airway microbiota is the mediator between respiratory infections and subsequent development of asthma.

## Conclusions and future directions

In conclusion, the current literature suggests a role of the microbiome in asthma and atopic disease development, with particular emphasis on early life dysbiosis. Notably, recent studies have identified shifts in specific bacterial genera and species, which could ultimately be applied as probiotic interventions prior to the development of asthma. These probiotic interventions could be given directly to the baby in early infancy once all safety concerns have been addressed. Another option for colonizing the infant is through maternal exposure to these microbes either before or after delivery. Prior to establishing these probiotic regimens however, shifts in early life gut and airway microbes could also be applied as microbe-based diagnostics to identify children at the highest risk of developing asthma and related allergic diseases.

Before any of these preventative or diagnostic techniques can be applied, future research should focus on validating the current findings in additional longitudinal human cohorts and improving humanized microbiome mouse models of airway and lung inflammation to mechanistically characterize the microbe-immune cell interactions promoting or protecting against asthma and atopic disease. Additionally, targeted-metabolomic and shotgun metagenomic sequencing strategies using stool, urine, and potentially breast-milk samples in human cohorts will better characterize the functional roles of these specific taxa in infant immune development.

Further, to better elucidate this early life critical window in humans, additional longitudinal cohorts should begin stool sample collection beginning at birth and continuing up to age 1 year (with at least bi-weekly collection points within the first 3-montths of life). Additionally, the collection of additional biological samples (namely blood and urine) during the first 3-months of life (though this is not often feasible) would be ideal to determine whether these gut microbial alterations occur prior to immune-dysregulation or vice versa.

Moreover, although this review focuses on the bacterial microbiome in asthma, there are many other microbial organisms (fungi and other eukarya, and viruses) that also play key roles in host physiology and immune development [6, 76–79]. Also, with the characterization of other microbiomes within the human body (i.e. placental, blood, breast-milk), it is likely that we will identify even more microbial taxa that are associated with airway diseases. As discussed in the previous sections, there is evidence in mice that asthma is a developmental origin disease, mediated by maternal gut microbial alterations in utero [56]. Thus it will be important to incorporate multi-biome analyses to potentially identify: (i) how children are being colonized with specific asthma related microbes; and (ii) roles of other microbial taxa in the pathogenesis of asthma and atopic disease. Ultimately however, this literature review presents research with promising future directions, offering an exciting outlook for future microbe-based preventative treatments and diagnostic strategies for asthma and atopic disease in children.

## Abbreviations

GF: germ-free
OVA: ovalbumin
HDM: house-dust mite
IgE: immunoglobulin-E
LPS: lipopolysaccharide
PAMP: pathogen-associated molecular pattern
TLR: toll-like receptor
MLN: mesenteric lymph node
SCFA: short chain fatty acid
DC: dendritic cell
iNKT cell: invariant natural killer T cell
CHILD Study: Canadian Healthy Infant Longitudinal Development Study
NP: nasopharyngeal
PD-L1: programmed death ligand-1
Th: T-helper
T-reg: T-regulatory
FLVR: *Faecalibacterium, Lachnospira, Veillonella, Rothia*
L/C: *Lachnospira/C. neonatale*
